# Establishing Trailer Ventilation (Boarding) Requirements for Finishing Pigs during Transport

**DOI:** 10.3390/ani4030515

**Published:** 2014-08-19

**Authors:** John McGlone, Avi Sapkota, Anna Johnson, Rebecca Kephart

**Affiliations:** 1 Animal and Food Sciences, Texas Tech University, Lubbock, TX 79409, USA; E-Mail: asapkota@purdue.edu; 2 Department of Animal Science, Iowa State University, Ames, IA 50011, USA; E-Mails: johnsona@iastate.edu (A.J.); rkdavis@iastate.edu (R.K.)

**Keywords:** boarding, pig, transportation, ventilation, welfare

## Abstract

**Simple Summary:**

Transport is an inevitable process in the modern swine industry due to the multiple-site approach to raising pigs and transport can be a significant source of stress to the animals, which raises a welfare concern. Maintaining the environment inside the transport trailer is crucial for pig comfort. This study aims to determine the amount of ventilation, or varied side-wall boarding, required to keep pigs within their thermal comfort zone. Examination of 302 trailers transporting 48,143 pigs found that pig losses were highest when low boarding levels (open sides) were used in cold air temperatures (<5 °C). In mild air temperatures (5 to 26 °C), boarding levels had little impact on pig losses.

**Abstract:**

Specifically, this study aimed to establish the effects on mortality and morbidity of boarding levels (amount of side-wall trailer ventilation) for finishing pigs in mild weather (8.80 ± 0.30 °C, 71.70% ± 1.12% humidity). Pigs from commercial finishing sites were transported in 302 pot-bellied trailers to commercial processing plants. Measures collected at the processing plant were rates of dead on arrival (DOA), non-ambulatory, non-injured (NANI), non-ambulatory, injured (NAI), and total dead and down (D&D). Boarding levels (% that side walls were closed off with inserted boards) were divided into 3 bins: low, medium, and high, and outside temperature was divided into 4 bins <5 °C, 5.10–10 °C, and 10.10–15 °C and >15 °C. Average rates of DOA, NANI, NAI, and D&D were approximately 0.30%, 0.12%, 0.04%, and 0.46%, respectively. The D&D was highest when boarding level was low with temperatures <5 °C (*p* < 0.05). However, variations in boarding level (medium and high boarding) in the temperature range of 5.10 °C to 23.30 °C did not affect pig losses.

## 1. Introduction

Transportation has become an inevitable aspect of pork production in the U.S., with more than 113 million pigs transported annually [1]. In North America, trailers are used for commercial transport, resulting in a known multi-factorial stressor for swine. Transit losses may be influenced by: trailer micro-environment, loading density, transit time, wait time both at the farm (from loading to leaving) and plant (prior to unloading), and handling procedures [2,3]. Trailer microenvironment is characterized by temperature, humidity, air speed, air pressure, level of gases (ammonia, carbon dioxide), and the amount and condition of any bedding [4]. Pigs do not have an efficient method of coping with thermal stress [5,6,7]; therefore, external cooling methods are necessary while transporting pigs to maintain their optimal thermal comfort zone. Trailers used for commercial transport are not equipped with artificial ventilation, but rather rely on passive ventilation of air in and out of side vents to circulate air within the trailer. The Transport Quality Assurance (TQA) handbook was released in 2002 to provide guidelines for many aspects of swine transport [8]. As new information is released, the handbook is updated, with the most recent edition being published in 2008. The TQA handbook provides the following guidelines for side vent positioning during transport: 10% of air vents should be open when air temperature is <−12 °C; 25% at −12 to −7 °C; 50% at −6 to 4.40 °C; 75% at 4.50 to 9.40 °C; and 100% at >9.4 °C. These suggestions are based on professional judgment and experience, rather than scientific data. Boarding refers to the amount that the side walls of the trailer were closed by inserting boards. Zero boarding would be a trailer with no added boards and the sides as open as possible. Boarding at 100% would mean all air spaces were covered with boards. This study aimed to provide science-based suggestions for boarding at various levels during transport of finishing pigs in mild weather.

## 2. Experimental Section

### 2.1. General

The experimental protocol for this study was approved by Institutional Animal Care and Use Committee at Texas Tech University and Iowa State University. The study took place during the spring and fall of 2012. Gilts and barrows (*n* = 48,143) used in this study were of mixed commercial genetics and raised in commercial finishing sites in the Midwestern U.S., then transported to processing plants in 302 pot-bellied trailers. A power test performed by Lewis [9] shows that 250 trailers will yield significant results in this type of study. Approximately 51 different drivers participated in the study; with some drivers hauling multiple loads. The average number of finishing pigs per trailer was 158.40 ± 1.06. Trailers were bedded with wood shavings (1 bale = 22.70 kg, 0.20 m^3^). Bedding level varied from 2 to 10 bales based on drivers’ discretion. Observations were made as pigs were unloaded at the processing plants. The 2 participating processing plants wish to remain anonymous.

This study was a part of a multi-year series of experiments with many experiments. Details of trailers and general conditions of the study are found in another paper in this series [10]. Briefly, the work was conducted at 2 commercial abattoirs and over 1000 pig finishing barns and sites that feed these plants in the Midwestern USA. Trucks pulled trailers that were primarily pot-belly designs described in other papers [2,10]. Transport times ranged from 30 min to 12 h and were used as a covariate but were found to not impact our findings. Bedding levels were held constant. Most trailers contained mixes of barrows and gilts (so sex effects cannot be examined). Likewise, extremes in air temperature were not examined because boarding is held constant in cold weather (near 100% boarded) and in warm weather (no boarding, or 0% boarded). Therefore, this dataset is only during relatively mild air temperatures (not during cold or heat stress; see below). We know from our other work [10] that pig surface temperature varies little in the relatively mild conditions of this study. Finally, driver effects could not be evaluated because most data points were from a single driver and therefore a single experimental unit. We did not have the opportunity to evaluate replications of drivers with several boarding levels.

### 2.2. Treatments

Outside air temperature was logically divided into 4 bins: <5 °C, 5.1 to 10 °C, 10.10 to 15 °C, and >15 °C. Temperature ranges for <5 °C were −0.6 °C to 5.0 °C. Temperature ranges for >15 °C were 15.1 °C to 26.6 °C. Boarding levels, or the percent of plugs/panels used to close vents in the trailer, were divided into 3 bins: low (0–30%), medium (31–60%), and high (>61%). Higher boarding level percentages signify that fewer vents were left open. Table 1 shows the number of trailers with each boarding level across all temperature bins. Different boarding levels cause different levels of ventilation within the trailer, although we do not know how internal air speed changes with different boarding levels. In addition, boarding levels change the physical nature of the side panels and they change the insulation level of the side walls. Still, enough information on boarding levels is provided so others may replicate and extend this work in the future.

**Table 1:** Number of trailers represented in each boarding level by temperature bin.

Temperature	<5 °C			5.1 to 10 °C			10.1 to 15 °C			>15.1 °C		
Boarding level	Low	Med	High	Low	Med	High	Low	Med	High	Low	Med	High
*n* trailers	8	27	25	25	81	27	16	30	11	22	21	2

Drivers were requested to randomly assign boarding levels on each trailer at loading; these levels were confirmed by researchers when the truck arrived at the processing plant. Because of welfare concerns regarding low boarding levels in cold temperatures and high boarding levels in hot temperatures, these bins have smaller sample sizes than the others. Boarding levels given are representative of the boarding level in all compartments of the trailer. The researchers also tallied the number of transportation losses, confirmed the number of pigs on each trailer, and compared these numbers with plant records for quality control purposes.

Transport losses are classified as one of the following [11]:

- Dead on arrival (DOA): the animal dies during transit and arrives at the plant already deceased;
- Non-ambulatory, non-injured (NANI): the animal cannot move or walk, is fatigued, but does not show any obvious sign of injury, trauma, or disease; and may be standing, sitting or more commonly lying down; or
- Non-ambulatory, injured (NAI): the animal is injured during transport and shows obvious sign of injury, trauma, or disease;
- Total dead and down (D&D) is the sum of DOA, NANI, and NAI.

### 2.3. At the Processing Plant

Temperature and relative humidity (RH) were taken at the processing plants at the time of unloading (Kestrel 4500 Pocket Weather Tracker, Extech Instruments, Nashua, NH, USA). Because of personnel restrictions, temperature and RH were not recorded at the finishing site. Wait time at the plant was measured as the time between the arrival of the truck and the start of unloading. Trailers were unloaded in a random order depending on the preference of the driver. Order of unloading was not recorded. Skin surface temperature (SST) was measured on 5 random pigs selected from the first group and 5 random pigs selected from the second group. A random sample was considered as ignoring the first 10 pigs at the beginning of unloading, sampling 5 pigs within the next 50 unloaded (first group), ignoring another 10 pigs, and then sampling another 50 pigs (second group). Surface temperature was measured on the flank with an infrared thermometer with a sensitivity of 0.1 °C (Extech model 42570, Extech Instruments, Nashua, NH, USA).

### 2.4. Statistical Analyses

Primary model included effects of boarding level using trailers as experimental unit and outside air temperature and bedding level were covariates. All data were analyzed using SAS 9.2, using the General Linear Models procedures (SAS, 2010 SAS Inst., Cary, NC, USA). Average wait time, SST, and average rates of DOA, NANI, NAI, and D&D were calculated. Rates of losses in each temperature bin for each boarding level were calculated and least square means were compared in each bin. When comparing the means, *p* values <0.05 were considered significant.

## 3. Results and Discussion

Average air temperature at the plant was 8.80 ± 0.30 °C (−0.60 to 23.30 °C) and average RH was 71.70 ± 1.12% (19 to 100%). Average wait time at the plant for the trailers was 43.70 ± 1.74 min (range: 0 to 161 min; *n* = 234). Average skin surface temperature on the flanks of the pigs was 25.20 ± 0.22 °C (17.30 to 32.50 °C; *n* = 219).

Rates of DOA, NANI and NAI were not affected by boarding level when outside air temperature and bedding levels were used as covariates (*p* > 0.05); therefore, these factors were not considered in further data analysis. Average rates of DOA, NANI, NAI and D&D per trailer were 0.30 ± 0.04%, 0.04 ± 0.01%, 0.12 ± 0.02%, and 0.46 ± 0.05% respectively.

**Figure 1:**
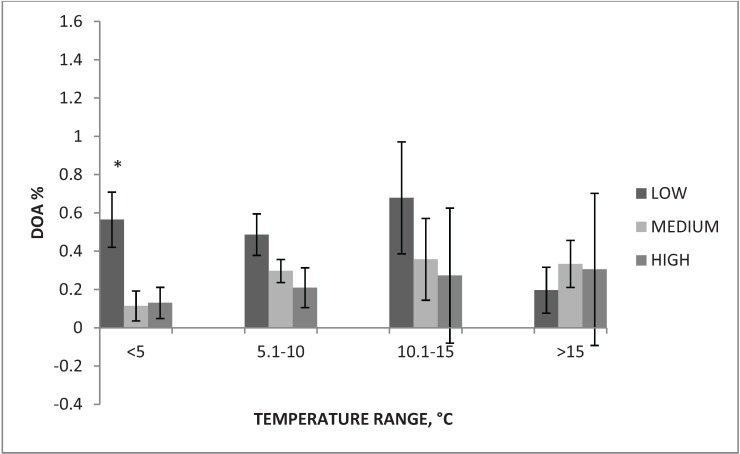
Rate of dead on arrival (DOA) for each boarding level ^1^ across all temperature bins.

When pigs were transported at air temperature <5 °C and low level of boarding was used, rate of DOA was higher (0.57 ± 0.14%), compared to medium (0.11 ± 0.08%) and high (0.13 ± 0.08%), (*p* < 0.05). Figure 1 shows the rate of DOA across all temperature bins by boarding level.

* *p* < 0.05 within temperature bin. ^1^ Low: boarding level <30%; medium: boarding level =31% to 60%; high: boarding level >60%.

**Figure 2:**
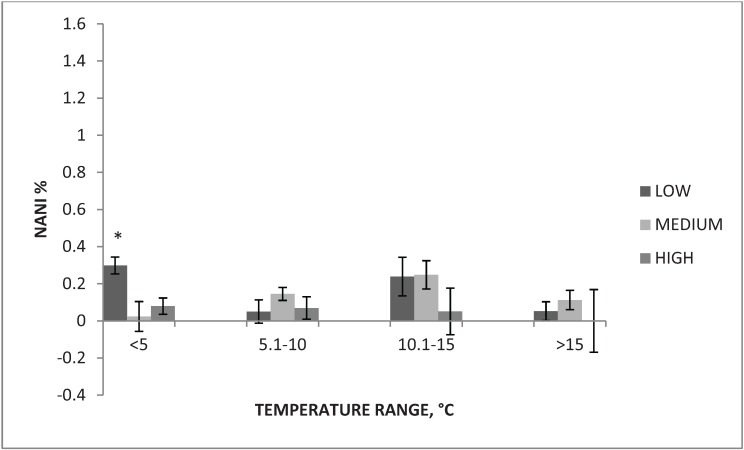
Rate of non-ambulatory, non-injured (NANI) for each boarding level ^1^ across all temperature bins.

Similar patterns were found for rates of D&D and NANI. Average rates of NANI at air temperature <5 °C while using low, medium and high boarding levels were 0.30% ± 0.05%, 0.02% ± 0.08% and 0.08% ± 0.04%, respectively. Figure 2 represents the rates of NANI by boarding level for each temperature bin.

**Figure 3:**
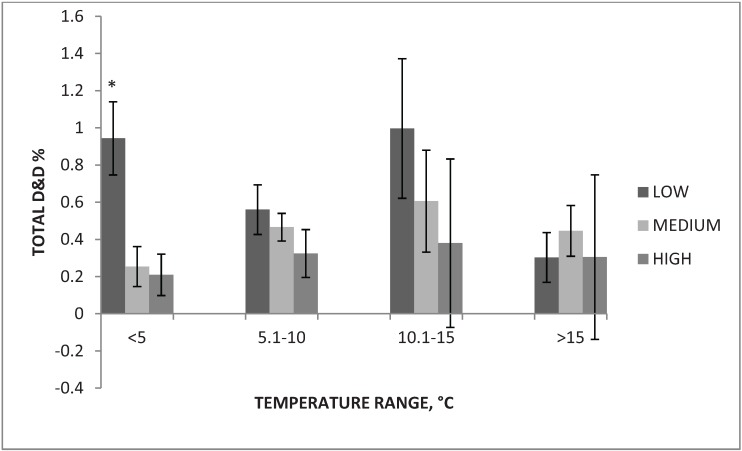
Rate of total dead and down (D&D) for each boarding level across all temperature bins.

Rates of D&D at air temperature <5 °C while using low, medium and high boarding levels were 0.94% ± 0.20%, 0.25% ± 0.11% and 0.21% ± 0.11%, respectively. Figure 3 represents D&D for each boarding level across all temperature bins.

Overall, low boarding levels resulted in the highest percentage of D&D across all temperature bins except for >15 °C. During transport in these colder temperatures, the air moving into the trailer may result in the pigs becoming too cold, especially if the bedding level is not appropriate. Too much bedding can accumulate moisture, which combined with cold air, may result in frostbite [10]. Another factor may be that the pigs exhaust themselves from shivering to maintain body heat. This study agrees with the TQA handbook’s guidelines for boarding requirements during transport of swine based on the finding that between 5 °C and 15 °C, boarding levels of 25%, 50%, or 75% produced statistically similar levels of NANI and DOA.

* *p* < 0.05 within temperature bin. ^1^ Low: boarding level <30%; medium: boarding level 31% to 60%; high: boarding level >60%.

* *p* < 0.05 within temperature bin. ^1^ Low: boarding level <30%; medium: boarding level 31% to 60%; high: boarding level >60%.

Total transport losses are estimated to cost the US pork industry $50 to $100 million annually [12]. This figure includes direct cost due to loss of pigs and indirect costs due to labor and facilities required to handle D&D pigs. The same review estimates that annual DOA, NANI, NAI, and D&D rates average 0.25%, 0.37%, 0.05%, and 0.69%, respectively [11]. It should be noted that the results of this study show a higher proportion of NAI compared to NANI. Some plants do not differentiate between NAI and NANI, but rather list all non-ambulatory animals as “total down”. These rates vary according to environmental conditions [2], management practices [13,14] genetic line [15,16], and transport facilities [17]. These figures represent not only a financial loss, but also an animal welfare concern. Sutherland [2] showed that rates of DOA increased at temperatures above 20 °C and rates of NANI increased as the temperature dropped below 5 °C. The findings of the present study agree with Sutherland’s NANI finding [2]. In the same study, waiting time at the plant influenced rates of DOA, NANI, and D&D; DOA and D&D were highest when wait time exceeded 4 h. In the current study, wait time did not have an effect on losses, which may be because wait time in this study ranged from 0 to 161 min. In Sutherland’s study, there was no difference in transport losses within this wait time range.

## 4. Conclusions

This study revealed that at outside air temperatures <5 °C, when less boarding was used average rates of DOA, NANI, and D&D increased. Below 5 °C, boarding levels should be above 30%. This indicates poor animal welfare and high economic loss. Using medium or high boarding levels at cold temperatures can reduce transportation losses. Boarding level should increase as outside air temperature decreases. Based on these results, the boarding level recommendations outlined in the TQA handbook are adequate for swine transport. Further evaluation needs to be done regarding proper ventilation in individual compartments and different types of trailers [18]. Also, this study needs to be conducted in different climates, such as the northeast U.S. Trailers with artificial ventilation are currently utilized in European countries, and still need to be tested in the U.S. [19].
